# Radiomics of Lung Nodules: A Multi-Institutional Study of Robustness and Agreement of Quantitative Imaging Features

**DOI:** 10.18383/j.tom.2016.00235

**Published:** 2016-12

**Authors:** Jayashree Kalpathy-Cramer, Artem Mamomov, Binsheng Zhao, Lin Lu, Dmitry Cherezov, Sandy Napel, Sebastian Echegaray, Daniel Rubin, Michael McNitt-Gray, Pechin Lo, Jessica C. Sieren, Johanna Uthoff, Samantha K. N. Dilger, Brandan Driscoll, Ivan Yeung, Lubomir Hadjiiski, Kenny Cha, Yoganand Balagurunathan, Robert Gillies, Dmitry Goldgof

**Affiliations:** 1 Massachusetts General Hospital, Boston, Massachusetts;; 2 Columbia University Medical Center, New York, New York;; 3 University of South Florida, Tampa, Florida;; 4 Stanford University, Stanford, California;; 5 University of California Los Angeles, Los Angeles, California;; 6 University of Iowa, Iowa City, Iowa;; 7 Princess Margaret Cancer Center, Toronto, Ontario, Canada;; 8 University of Michigan, Ann Arbor, Michigan; and; 9 Moffitt Cancer Center, Tampa, Florida

**Keywords:** radiomics, reproducibility, imaging features, lung cancer

## Abstract

Radiomics is to provide quantitative descriptors of normal and abnormal tissues during classification and prediction tasks in radiology and oncology. Quantitative Imaging Network members are developing radiomic “feature” sets to characterize tumors, in general, the size, shape, texture, intensity, margin, and other aspects of the imaging features of nodules and lesions. Efforts are ongoing for developing an ontology to describe radiomic features for lung nodules, with the main classes consisting of size, local and global shape descriptors, margin, intensity, and texture-based features, which are based on wavelets, Laplacian of Gaussians, Law's features, gray-level co-occurrence matrices, and run-length features. The purpose of this study is to investigate the sensitivity of quantitative descriptors of pulmonary nodules to segmentations and to illustrate comparisons across different feature types and features computed by different implementations of feature extraction algorithms. We calculated the concordance correlation coefficients of the features as a measure of their stability with the underlying segmentation; 68% of the 830 features in this study had a concordance CC of ≥0.75. Pairwise correlation coefficients between pairs of features were used to uncover associations between features, particularly as measured by different participants. A graphical model approach was used to enumerate the number of uncorrelated feature groups at given thresholds of correlation. At a threshold of 0.75 and 0.95, there were 75 and 246 subgroups, respectively, providing a measure for the features' redundancy.

## Introduction

Radiomics, “the high-throughput extraction of large amounts of image features from radiographic images” (1), has been used to provide quantitative descriptors of normal and abnormal tissues during classification and prediction tasks in radiology and oncology. Currently, several groups within the Quantitative Imaging Network (QIN) are developing radiomic “feature” sets to characterize tumors. These mathematical descriptors provide ways to characterize the size, shape, texture, intensity, margin, and other aspects of the imaging features of nodules and lesions, with the eventual goal of being able to separate benign from malignant nodules, assessing response to therapy, and correlating imaging with genomics. Because tumors usually occupy a relatively small portion of the radiological image or volume, a common requirement for any feature extraction algorithm is dependence on a provided region of interest (ROI), commonly referred to as segmentation.

The purpose of this study was 2-fold as follows:

(1) To investigate the sensitivity of quantitative descriptors of pulmonary nodules to segmentations by examining the variability of the features to variations in the segmentations caused by use of different algorithms and/or initial conditions they require.
(2) To illustrate comparisons across different feature types and features computed by different implementations of feature extraction algorithms.

This important first step is required to understand feature stability and associations among features. However, because of the nature of the data available, in this work, we could not and do not answer questions about the utility of specific quantitative image features for prediction of malignancy, pathological nodule diagnosis, response to therapy, or other possible clinically related questions.

The images used in this study were generated during a previous multisite study (2) that investigated the repeatability and reproducibility of lung nodule segmentation algorithms in a data set of 52 lesions in 41 computed tomography (CT) volumes, each of which was segmented by 3 different algorithms. All CT volumes and segmentations are available in The Cancer Imaging Archive (TCIA) as a shared list for easy distribution. Here, 8 sites participated and 7 provided the features extracted at each site using the aforementioned segmentations and the “dictionary” files that provided metadata about the features they computed, whereas 1 site provided the infrastructure and statistical analysis support for the project. Efforts are underway to create an ontology of features based on the information provided in these feature dictionaries.

## Methods

### Data Set

We used 41 CT volumes from 5 collections of Digital Imaging and Communications in Medicine (DICOM) CT images of patients with non-small cell lung cancer and a thoracic phantom previously used for the QIN lung segmentation challenge (2) from the following sources: CUMC_FDA Phantom, Moffitt Cancer Center, the Reference Image Database to Evaluate Therapy Response (RIDER) (3), Stanford University Medical Center, and the Lung Image Database Consortium (LIDC) (4). All image data from human subjects were previously deidentified under institutional review board protocols in place at participating institutions before deployment on TCIA (5); therefore, for the purposes of the current study, these data are not considered as human subjects' data and, hence, no additional institutional review board supervision is required. The collection consisted of lung CT volumes collectively containing 52 nodules and segmentations. In total, 40 patients with cancer with a single lesion of interest per scan and 12 phantom nodules all within 1 additional scan were included. Nodules varied by location, size, and other attributes. Three different segmentation algorithms were used, with each using 3 different initial parameters (such as seed point or ROI), to obtain 9 segmentations per nodule, resulting in a total of 468 segmentations.

Here, 7 groups (University of Columbia, Moffitt Cancer Center/University of South Florida, Stanford University, University of California Los Angeles, University of Iowa, Princess Margaret Cancer Center, and University of Michigan) obtained the data set, including all images and segmentations, from TCIA (5), and each computed their own set of features for each of 468 segmentations and uploaded them to a Web site set up for this feature challenge. In addition, each participant uploaded a “feature dictionary” that facilitated mapping of each feature to one of a set of predefined feature categories and provided other metadata about the features including a short description.

### Feature Ontology

One of the ongoing goals for this project is to define an ontology of features that are typically extracted for lung lesions. All participants, through an iterative process, defined a set of feature classes that covered the types of features that were being extracted by this group. Some of the feature classes (eg, texture) were further subdivided into feature subclasses. Participants provided information about the feature “class” and “subclass” as part of the dictionary file.

This ontology also facilitated comparisons of features across institutions within a class or subclass. The following feature classes were agreed upon: size, intensity, global shape descriptors (GSDs), local shape descriptors (LSDs), and margin and texture features. Because of the diversity of texture features, this class was further divided into several subclasses of texture features such as gray-level co-occurrence matrix (GLCM) features (also known as Haralick texture features), Laplacian of Gaussian features, Law's texture features, run-length features, and wavelet-based features. When sites provided their feature dictionary, they described each feature in terms of its name, class, subclass (if applicable), and some general descriptors relating to the calculation of the feature such as whether the calculation was either 2 dimensional (2D) or 3 dimensional (3D), whether it was multiscale, and, if yes, the number of scales. Each participating site provided this dictionary; a brief description of their submitted features including the computational pipeline is provided below.

### Feature Computation

The extractions for features of lung lesion typically followed a standard sequence of image analysis steps. Although nodule segmentations were provided, some algorithms additionally segmented the surrounding parenchyma and/or the lungs. Preprocessing steps varied among participants, and included steps such as upsampling the volume to high-resolution isotropic spaces and/or image cropping.

Following any preprocessing steps, typical features computed included those related to the size of the lesion (eg, volume, maximal diameter, and size of bounding box), local (eg, roughness) and global (eg, eccentricity) shape descriptors, lesion intensity (eg, average, median, maximum, minimum, and standard deviation voxel values), margin (eg, edge gradients and surface normals), and texture (eg, those based on GLCMs and wavelets).

Some features (eg, volume, maximal diameter, and shape) were calculated on the basis of only the provided nodule segmentation, whereas others were based on the intensity values within the segmentations of the CT volume. Some participants' only 3D features were calculated, whereas for some, both 2D (for example, from the maximal transaxial cross section of the provided segmentation) and 3D features were calculated. Some features (eg, textures) were calculated at each point within the segmentation and aggregated over the volume. Some of these features were calculated at multiple scales and directions, whereas others were scale- and rotation-invariant. The aggregation methods varied among the participants from simple averaging over scales, directions, and locations to more complicated methods such as kernel functions or Gaussian mixtures.

In aggregate, 7 participating sites (Table 1) provided a total of 830 features. The following is a brief summary of the processing used by each participating institution along with, in some cases, references to more complete descriptions of the features.

**Table 1.** Number of Features per Type Submitted by Each Participating Institution

Participant	Size	GSDs	LSDs	Intensity	Margin	Texture					Total
						GLCM	LoG	Law's	Run Length	Wavelet	
CUMC	3	4	8	5		17	6	14		14	71
PM	3	2		5							10
Stanford	2	1	78	17	27	72					197
UCLA	1			4		10					15
UIowa	2	6		9	151			136			304
UMICH	4	5		6	18				16		49
USF	5					6		125	20	28	184
Total	20	18	86	46	196	104	6	275	36	42	830

Abbreviations: CUMC, Columbia University Medical Center; PM, Princess Margaret Cancer Center; UCLA, University of California Los Angeles; UIowa, University of Iowa; UMICH, University of Michigan; USF, University of South Florida.

### Columbia University Medical Center

The Columbia University Medical Center feature pipeline (6) comprised 2 stages—image preprocessing and feature extraction. In the image preprocessing stage, each nodule was first cropped out by using a bounding box extending 1 cm beyond the largest nodule extent in all 3 dimensions, and then linearly interpolated into 3D isotropic images with voxel spacing of 0.5 × 0.5 × 0.5 mm^3^. In the feature extraction stage, a set of 71 radiomic features were extracted. Among the 71 radiomic features, some were computed in 2D and some in 3D. The 2D features were calculated on the automatically determined axial image where the nodule had the maximal diameter. During implementation, when involving parameters of neighborhood, direction, and distance between pixels/voxels, 8 connected pixels were considered as the neighboring pixels for 2D analysis, whereas 26 connected voxels were considered as the neighboring voxels for 3D analysis; 8 directions were used for 2D analysis and 13 directions were used for 3D analysis; unless specified, the distance between 2 neighboring pixels/voxels was 1. The Columbia University Medical Center feature extraction algorithms were all written in MATLAB (MathWorks, Natick, MA).

### Princess Margaret Cancer Center

The Princess Margaret Cancer Centre extracted the 3D lesion by applying the mask to the 3D image set. The extracted image subset was used to calculate the intensity and size subclass features using in-house algorithms developed in Matlab. The only exception was that the 3D images were upsampled to 1-mm sections before being applied to the surface area algorithm which was also written in Matlab.

### Stanford University

Stanford University used a prototype of its 3D Quantitative Image Feature Pipeline (7) to compute 3D features from the 468 nodule segmentations. The software was written using Matlab, and a DICOM Segmentation Object, which unambiguously defines the volume of interest containing the nodule, and a DICOM Image Series, which consists of the CT sections acquired by scanning the patients, were considered as input. The Quantitative Image Feature Pipeline consists of a preprocessing stage, which establishes voxel-to-millimeter scaling factors and crops each nodule with a bounding box extending 2 cm beyond the largest nodule extent in all 3 dimensions to limit storage and processing requirements. It then computes the following general classes of features:

(1) Size features include surface area (mm^2^) and volume (mm^3^).
(2) Intensity features include various statistics of the voxel intensity histogram.
(3) GSDs include sphericity.
(4) LSDs include roughness statistics and local volume-invariant statistics.
(5) Margin features include statistics of a 2-parameter fit to sigmoid function of intensities along surface normals at 800 locations around the nodule.
(6) Texture features include mean and standard deviation over orientation of Haralick features (derived from the co-occurrence matrix) at 3 scales (1, 2, and 3 mm), for a total of 197 scalar-valued features.

Detailed formulae for these features can be found in the Stanford University Technical Report by Echegary (8).

### University of California Los Angeles

The University of California Los Angeles feature pipeline created the reported feature values by first reading in the nodule cases as DICOM images and the nodule ROIs from segmentations provided. For each nodule and each ROI, 15 features were calculated and submitted for analysis, which represented a modest subset of features available. Volume in cubic millimeter was calculated from the number of identified voxels in each ROI and the size of each voxel as reported in the DICOM header. Four additional features were based on the intensity distribution of voxels contained within each ROI including mean, standard deviation, skewness, and kurtosis. Finally, 10 features from the GLCM texture family were submitted. The co-occurrence matrices were formed using 32 quantization levels (of gray levels or Hounsield Units (HU) bins). The number of directions used (also referred to as offsets) were based on the direction on the 26-connectivity that is typically used in 3D, resulting in a total of 13 offsets (=26/2 because of symmetry). This results in a total of 13 co-occurrence matrices. The measures calculated (contrast, dissimilarity, homogeneity, energy, and entropy were used for this study) were obtained for each co-occurrence matrix formed, and the final feature values submitted were the mean value and range value (maximum − minimum) of each measure over the 13 co-occurrence matrices.

### University of Iowa

The University of Iowa feature pipeline consisted of image preprocessing and feature extraction, which were both performed using scripts written in Matlab. The University of Iowa approach included feature extraction from both the lung nodule and the surrounding lung parenchyma. For this study, the nodule segmentation mask was provided, from which the maximum radius of the nodule was determined. Extending from the nodule boundary, all voxels within a distance of the maximum nodule radius were included in the parenchyma mask. If nonvalid parenchyma regions were present, such as pleural wall, they were manually excluded from the parenchyma mask. In the feature extraction stage, a set of 304 radiomic features were calculated as previously described (9). From the nodule, 159 features were extracted including intensity, size, shape, and texture. From the surrounding parenchyma mask, 145 features were extracted including intensity and texture features, which are reported here as margin features. Further, 2D and 3D shape and size features were calculated using physical distance, to compensate for voxel resolution differences in the data set. Texture features were calculated based on a 3D, rotation-invariant implementation of Law's texture energy measures (10) aggregated as histogram summary statistics.

### University of Michigan

The general Michigan feature extraction pipeline consists of 3 stages—preprocessing, nodule segmentation, and feature extraction. It was developed using C language, and it takes DICOM images as input. In the preprocessing stage, a lung mask is first generated by segmenting the lungs using k-means clustering. By using an input bounding box around the target nodule (with a margin of about 10 mm around the nodule), a volume of interest containing the nodule is extracted. An image of isotropic resolution is obtained by performing linear interpolation in x, y, and z directions. The nodule segmentation stage (stage 2) was not activated in this specific challenge because the nodule segmentations were provided and they were imported into the pipeline. The imported nodule segmentations were positioned on the interpolated sections by selecting the nodule segmentation nearest to each interpolated section. In the feature extraction stage (stage 3), the size, intensity, texture, shape, and margin features were extracted. The mediastinal or pleural voxels were excluded from the feature extraction process using the lung mask. Within the segmented regions, size features, such as volume, surface area, perimeter, and diameter, and intensity features, including average, variance, skewness, and kurtosis of the gray-level histogram, were extracted (11). After performing the rubber band straightening transform (12) of the segmented nodules section by section, texture features based on run-length statistics (run-length features) are extracted from the Sobel-filtered rubber band straightening transform images (11). In addition, margin features based on the statistics of gradient field strength and orientation on spherical shells around the nodule surface were extracted to describe the sharpness of the nodule boundary and the smoothness of the nodule margin (13, 14). Finally, global shape descriptors based on the statistics of nodule radii were extracted as descriptors of the irregularity of the nodule shape (13).

### Moffitt Cancer Center/University of South Florida

The Moffitt Cancer Center/University of South Florida (USF) team's workflow was implemented on custom implementations of imaging features in C/C++ language that could be called from a commercial imaging workstation (15). Quantitative imaging features were extracted on these ROIs. The USF pipeline has only 1 stage. There were no preprocessing operations applied on the DICOM images or the segmentations. The in-plane pixel spacing parameters are typically defined during the scan, which is dependent on the patient size and/or the scan center. The USF methods had no modifications applied to either segmentation or DICOM files. The implementation had 184 features in total, categorized into size, density intensity, shape, margin, and texture (co-occurrence, run-length, wavelets, and Law's features). All the features were computed in 3D. These features were qualified for their reproducibility on a test–retest data set (16). These features have been shown to be effective for predicting malignancy in screening setting (17) and as an indicator of disease progression and related to the genome (18, 19).

### Statistical Analysis

Our first goal was to understand the sensitivity of the features to the segmentation. We used the repeated-measures concordance correlation coefficient (CCC) (20) as our statistical estimate of the repeatability and reproducibility of our features to the segmentations. We calculated intersegmentation algorithm, intrasegmentation algorithm, and total (combining intra- and inter-) CCC for each feature.

Our second goal was to understand the correlations among features collected by the 7 participants. For this, we calculated the association using the correlation coefficients (CCs) between all pairs of features. We expect similarly named features (eg, volume) to be highly correlated across different participant's implementations. However, we were also interested in observing correlations within feature sets provided by each participating institution and identifying unique, uncorrelated features both within and across all participating institutions.

A graphical model approach was used to examine the correlations among features. Each of the 830 features was modeled as a node in an undirected graph, and the edge weight between 2 nodes was the absolute value of the CC. We used both the Pearson and Spearman CCs, as associations between features can be either linear or nonlinear (21, 22). Starting with a fully connected graph, edges were then filtered such that edges with weights less than the given threshold (T) were removed. This produced sets of disjointed subgraphs. We computed the number of such subgraphs (at least 1 node) for numerous different thresholds as a measure of the uniqueness of the features submitted.

## Results

### Feature Ontology

The number of features from each participant varied from 10 to 304, and Table 1 shows the features in each class and subclass as per the ontology used for this project, based on the metadata provided with each submission.

### Data Visualization

We have a developed a web-based interactive results viewer (see Supplemental Appendix) as part of the platform used for this challenge. This cross-browser tool, developed using JavaScript, allows users to explore a range of analyses of these data. Visualizations include histograms of features by site and feature class, heat maps of correlations of features between sites, and graphical models of the connectivity of features.

### Statistical Analysis

The first set of analyses was performed to estimate the robustness of features with respect to segmentation. We calculated the inter-CCC, intra-CCC, and overall CCC for each feature. High CCC imply that the features are not very sensitive to the underlying segmentation, whereas low CCC suggests that the characteristics of the underlying segmentation have a strong influence on the value of those features. The inter-CCC is an estimate of the stability of the feature across different segmentation algorithms, whereas the intra-CCC is the estimate of the stability of the feature within multiple segmentations of the same segmentation algorithm (with different initializations). In our previous work (2), we showed that the segmentations had higher repeatability than reproducibility (ie, were more similar among different runs of the same algorithm compared with those of different algorithms).

**Figure 1.**
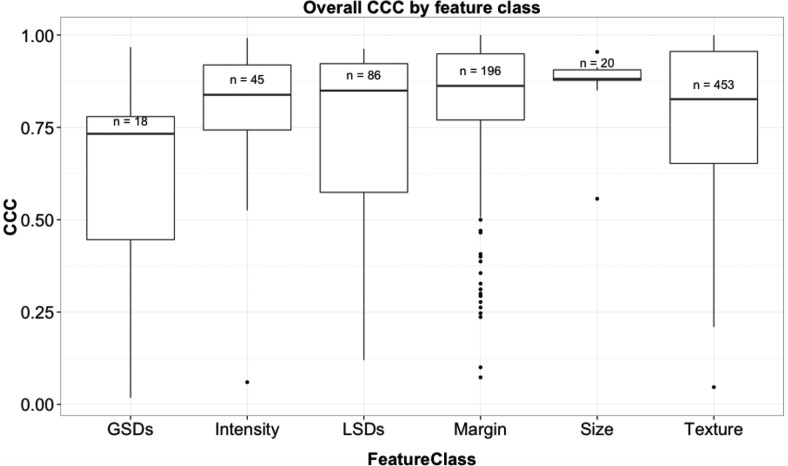
Overall concordance correlation coefficient (CCC) by feature class indicates the relative robustness of features to underlying segmentation.

**Figure 2.**
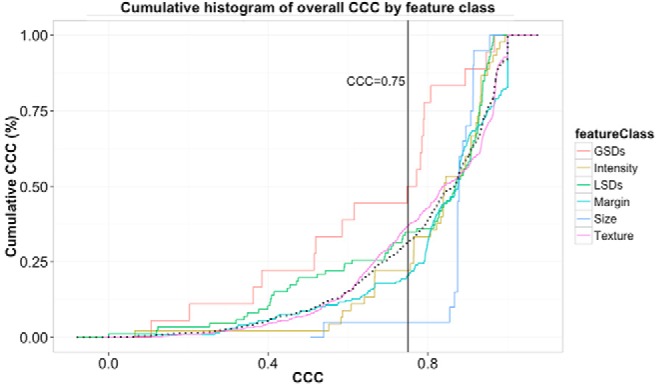
Cumulative histogram of overall CCC by feature class.

Figures 1 and 2 display the results of the analysis of feature stability to segmentation. Figure 1 displays the range of overall CCC for each feature class and shows the relative stability of feature classes to variations in segmentation algorithms. Clearly, the 18 features in the GSD class have the largest range of CCCs, whereas the 20 features in the size class have the smallest range. An ANOVA of the inter-CCC, intra-CCC, and overall CCC indicated that the CCC is different by feature class (*P* = .000125 for overall CCC, *P* ≪ .05 for inter-CC, and *P* = .0395 for intra-CCC). Further, post hoc comparisons based on the Tukey-HSD statistic suggested that the inter-CCC and overall GSD CCCs are lower and different from the other classes at an adjusted *P*-value of .05 for the inter-CCC and overall CCCs. Although we can make general observations about the sensitivity of features within a class to the underlying segmentation, there can be a considerable range of CCC values as seen on the width of the boxplots of the texture, GSD, and LSD classes.

Figure 2 displays cumulative density function of the overall CCC by feature class. In total, 68% of all features have an overall CCC of ≥0.75, whereas 24% of all features have an overall CCC of ≥0.95. However, only 50% of GSD features have a CCC of ≥0.75, whereas 95% of size features have a CCC of ≥0.75. In total, 63% of shape features, 78% of intensity features, 65% of LSDs, and 80% of margin features have a CCC of ≥0.75.

It is worth mentioning that although the boxplot in Figure 1 treats each feature as an independent measure, many features are expected to be either highly correlated or identical. For example, we would expect identical features (eg, volume) that are part of the feature pipelines of multiple participants to be highly correlated. Features within the same class (eg, GLCM features at different directions or scales) are also potentially correlated.

The second set of analyses was performed to compare and correlate features submitted by different groups. We calculated the CC for all pairs of features and validated the assumption that the same feature calculated by multiple participants would exhibit high correlation. The most obvious example of such a feature is the tumor volume, a member of the size class; as computed by 6 of the participants, the CCs between all pairs were between 0.9999 and 1. Other common features such as intensity-based mean, standard deviation, median, kurtosis, and skewness were calculated by many participants, and were highly correlated between many pairs of participants. Features from a similar class, such as texture features based on GLCMs, were also highly correlated among themselves.

**Figure 3.**
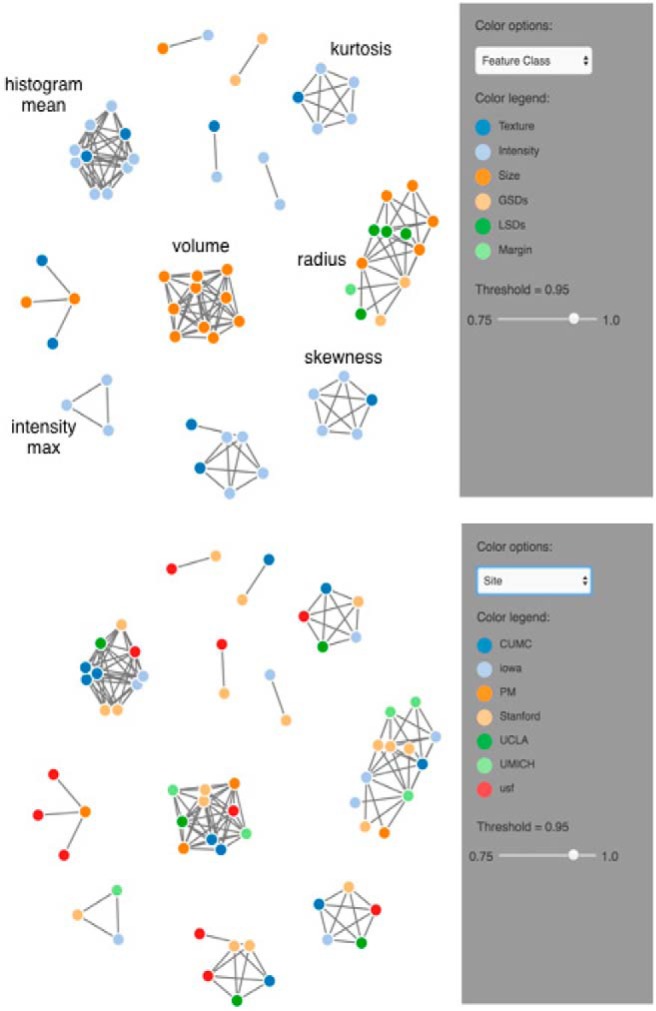
Example graphs of connectivity between feature nodes using a threshold of 0.95 for the correlation coefficient (CC) highlight correlation between features from different participants.

**Figure 4.**
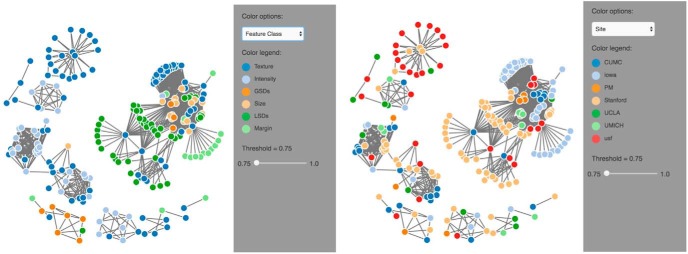
Graphical model of connected components at a CC of 0.75.

Next, we discuss the results from the graphical model approach to analyzing the correlations between features provided by different participants. Table 2 displays the number of noncorrelated subgroups as a function of the threshold of the CC used for connectedness. At a given threshold, each subgroup corresponds to correlated features, and the number of resulting subgraphs captures approximate dimensionality of overall features. We examined the composition of some exemplar connected subgraphs at different edge strengths. As expected, a lower threshold results in fewer subgroups, whereas a high threshold resulted in a large number of groups. Groups can be seen as a function of both participating institution and feature class in our interactive Web site, developed as part of our challenge platform. Figure 3 is an example of the graph at a threshold of 0.95, where the nodes are colored by a feature class (above) or a site (below). Only groups with >1 node are shown for clarity. Some subgroups consist of nodes from a single participant, whereas others have representation from many groups. Example groups that have representation from multiple participants include volume, radius, histogram mean, maximum, kurtosis, and skewness. Figure 4 displays the graph at a threshold of 0.75. As expected, the number of nodes per group is greater and with a more complex structure.

**Table 2.** Number of Non-Correlated Subgroups by Given Threshold and Correlation Type

Linear		Non-Linear	
T	No. of Subgroups	T	No. of Subgroups
0.75	75	0.75	58
0.80	103	0.80	80
0.85	150	0.85	120
0.90	245	0.90	172
0.95	382	0.95	246

T is the correlation threshold.

Six of the features in the skewness subgroup were related to the histogram skewness. However, 1 feature was the entropy mean, which, in turn, was connected to a run-length (subclass), texture (class) feature. This graphical tool allowed us to explore both expected and unexpected clusters (eg, features from different classes) of features at different levels of connectedness.

## Discussion

This study was designed to examine the sensitivity of quantitative image features to tumor segmentation and to study correlations between features computed by different implementations of feature extraction algorithms.

This was done by examining individual feature values produced by software instances from 7 independent participating institutions across 9 different image segmentation results for each of the 52 lung nodules in chest CT scans. This study does not address the ultimate utility of these feature values in predicting or assessing outcome measures such as assessing whether a lung lesion is either benign or malignant or whether the patient is responding to therapy. However, unless segmentation algorithms perform perfectly and reproducibly, it is important to understand the stability of any feature to segmentation that could be considered for prediction of these types of clinical variables.

However, in all of the above contexts, ROI identification (segmentation) becomes most critical. Stability of segmentation algorithms to perform reproducibly well becomes important. Our prior work has shown acceptable repeatability and reproducibility of segmentations across institutions (2). In continuation, understanding the variability of any feature derived on these segmentations becomes ever more critical, as the metrics are typically used to relate to the clinical variables and or track response to treatment.

Because of the variety of segmentation algorithms available and because of the variability of any nonfully automated algorithms, sensitivity to segmentation algorithm results is an important issue. However, it is recognized that this is not the only issue that needs to be addressed for understanding the ultimate utility of radiomic features; even features that can be shown to be stable over a wide range of segmentation results (eg, a feature that is a constant) may not necessarily be useful in performing a given outcome-related task such as differentiating benign nodules from malignant ones or in assessing response to therapy. Although we have not investigated utility in this manuscript, future studies of radiomic features should investigate both their robustness to segmentation and their usefulness in a predictive model, for example, differentiating between benign and malignant nodules in a lung screening CT setting or predicting progression-free survival from pretreatment images.

As expected, the results presented here did show high correlations between certain groups of features (eg, size features calculated across participants). However, there were also specific features within these groups that did not. One example was within the intensity feature category; although most participants reported the mean intensity (HU) of each lesion, some participants additionally reported unique and uncorrelated features, such as the size of airspaces within the lesion or the lesion's maximum or minimum intensity values. Although these features may be intensity-related, they may not be highly correlated to the mean intensity value of the total nodule, and therefore, it is not unexpected that these features would produce values that would be shown as outliers in a distribution of intensity features. That said, these features may contribute information that is complementary to the information provided by the other intensity features and may be able to contribute to the outcome-related task (eg, discrimination or assessment of response).

There were several lessons learned from this study. First, it showed that there is substantial value in comparing feature values among different groups, even when the feature values are expected to be the same or very similar. For example, by comparing lesion volume values across different lesions, segmentations and participants, we could uncover subtle differences and even errors in approach and calculations that may not have been discovered otherwise, including how participants were handling cases where the section thickness and section spacing were not the same value (eg, overlapping images).

In conclusion, this study also showed the value of using phantom images or synthetic images where there are objects with known values such as known density or known volume. These provide users the ability to gain confidence that their methods and calculations are performing in a manner similar to some reference method(s). These are helpful steps that can and should be taken when possible before moving on to more complex objects such as the lung lesions used in this study. In this study, having a common set of reference images, well-specified objects and existing object masks allowed the authors to focus on the very specific task of feature computation, its sensitivity to segmentation results, and the associations among specific features.

### Supplemental Materials

Supplemental Appendix 1:

Supplemental Appendix 2:
